# Phloridzin as a Nutraceutical for Cancer Prevention and Therapy: A Comprehensive Review of Its Mechanisms, Bioavailability Challenges and Future Applications

**DOI:** 10.1002/fsn3.70744

**Published:** 2025-08-04

**Authors:** Praveen Dhyani, Priyanka Sati, Dharam Chand Attri, Eshita Sharma, Ruchi Soni, Javad Sharifi‐Rad, Daniela Calina

**Affiliations:** ^1^ Institute for Integrated Natural Sciences University of Koblenz Koblenz Germany; ^2^ Department of Biotechnology Kumaun University Bhimtal India; ^3^ Department of Botany Central University of Jammu Rahya‐Suchani (Bagla) India; ^4^ Department of Molecular Biology and Biochemistry Guru Nanak Dev University Amritsar India; ^5^ Regional Centre for Organic and Natural Farming Ghaziabad India; ^6^ Universidad Espíritu Santo Samborondón Ecuador; ^7^ Centro de Estudios Tecnológicos y Universitarios del Golfo Veracruz Mexico; ^8^ Department of Medicine College of Medicine, Korea University Seoul Republic of Korea; ^9^ Department of Clinical Pharmacy University of Medicine and Pharmacy of Craiova Craiova Romania

**Keywords:** anticancer activity, bioavailability, flavonoids, GLUT inhibitors, phloridzin

## Abstract

The global rise in cancer incidence has driven the search for safer, more effective therapies, with natural compounds gaining increasing attention. Phloridzin, a dihydrochalcone glycoside abundant in apple trees (*Malus* spp.), has demonstrated notable anticancer properties. This review summarizes its pharmacological profile, natural sources, and structural characteristics, with a focus on its mechanisms of antitumor action. We conducted a structured literature search across SCOPUS, PubMed, Google Scholar, and TRIP databases, highlighting studies on phloridzin's anti‐proliferative, pro‐apoptotic, anti‐inflammatory, and metabolic regulatory effects across various in vitro and in vivo cancer models. Key mechanisms include glucose transporter inhibition (GLUT1/2), modulation of PI3K/AKT/mTOR and JAK2/STAT3 signaling, and suppression of metastasis and angiogenesis. Despite compelling preclinical evidence, phloridzin's clinical application is limited by low bioavailability. Novel delivery systems and synthetic derivatives, such as fatty acid esters, have shown improved pharmacokinetic profiles and efficacy. Future studies should prioritize translational research and clinical trials to validate phloridzin's potential as an adjunct or alternative therapy in oncology.

## Graphical abstract

Phloridzin, a bioactive flavonoid from Malus species, demonstrates diverse therapeutic effects including anticancer, antidiabetic, hepatoprotective, cardioprotective, neuroprotective, and antimicrobial activities. Its anticancer efficacy is mediated through glucose transporter inhibition and modulation of oncogenic signaling pathways. The compound shows promise as a multitarget nutraceutical, though limited by poor bioavailability that may be addressed through advanced formulations and derivatives.

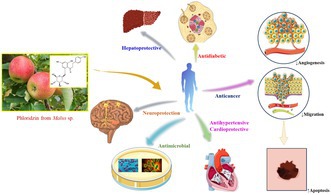

## 1 Introduction

Phytochemicals are biologically active organic substances found in plants, synthesized by the secondary metabolism of cells. Phytochemicals have a broad role in plant systems, such as (among many) color, aroma, flavor, and defense mechanism. For ages, these phytochemicals were vital components in traditional health care systems worldwide, as plants were one of their essential components. Nevertheless, in modern medicine, owing to their capability to regulate various physiological processes in humans with fewer side effects, they are widely researched and used as plant‐based drug molecules instead of synthetics (Tzevtkov et al. 2023). Among the various classes of phytochemicals, flavonoids have been studied in numerous clinical trials due to their multiple pharmacological activities beneficial to the human body (Bisol et al. 2020; Russo et al. 2019). Phloridzin is a flavonoid with diverse bio‐effects belonging to the subclass of dihydrochalcones. It is also known as phloretin 2′‐O‐glucoside, phlorizin, phloridzin, phlorhizin, or phlorizoside (Baldisserotto et al. 2012). Phloridzin is a potent phytochemical exhibiting various bioactivities, such as antihyperglycemic, anti‐oxidant, anti‐inflammatory, hepatoprotective effect, antibacterial, cardioprotective, and anti‐tumor (Mariadoss et al. 2019; Nakhate et al. 2022; Patel 2022; Trifan and Luca 2023). Notably, the antitumor activity of phloridzin, in turn, makes it a potent compound with significant potential in cancer prevention and treatment. It becomes more critical as cancer remains one of the most devastating diseases worldwide. The International Cancer Observatory estimates that 9.9 million people worldwide were affected by cancer in 2020. Moreover, the cases are projected to rise to 28.4 million new cancer cases in 2040, severely affecting specifically middle and low‐income countries (Sung et al. 2021). Typically, cancer is characterized by the uncontrolled growth and multiplication of cells within tissues, resulting in a mass called a tumor, which can expand throughout the affected organ or spread to other tissues and organs in the body.

(Jiang et al. 2015). In general, cancer treatment is a complex process due to the wide variety of cancer types, each presenting unique challenges and characteristics. Among the many critical issues, research and development of targeted therapies, as well as the discovery and application of novel agents or compounds, remain particularly demanding. Furthermore, conventional treatment methods—such as chemotherapy, radiation, and surgery—can still cause significant side effects, limiting their effectiveness and negatively impacting patients' well‐being (Yagawa et al. 2017). Radiotherapy and chemotherapy are related to side effects, ranging from mild gastrointestinal changes and nausea to severe gut mucosa dysfunction, cardiovascular toxicity, or immunity disorders (De Ruysscher et al. 2019), mainly because they do not differentiate between normal and cancer cells. More recent synthetic drugs, on the other hand, offer a more targeted and less toxic alternative to traditional chemotherapy; however, their efficacy varies depending on the type and stage of cancer. For instance, Cisplatin—a known radiosensitizer and chemotherapeutic agent—is used in the treatment of approximately 50% of cancer patients due to its multidirectional mechanism of action. Nevertheless, it is associated with broad side effects such as nephrotoxicity, ototoxicity, and gastrointestinal toxicity, along with the development of resistance during therapy (Ghosh 2019; Skowron et al. 2018). Another antineoplastic agent, 5‐fluorouracil, is commonly used to treat gastrointestinal tract, head and neck, and breast carcinoma. But at the same time, the toxicity induced by it may lead to ulceration of the gastrointestinal tract with symptoms of shortness of breath (Hodroj et al. 2021). Similarly, the existing popular drugs such as Doxorubicin, Cyclophosphamide, and Paclitaxel have the limitation of lack of selectivity, side effects, drug resistance, and dose limitation (Mustafa et al. 2023; Nagappa et al. 2014; Thorn et al. 2011). The limitations of conventional cancer therapies, including toxicity and resistance, underscore the urgent need for alternative, safer treatments. Phytochemicals—bioactive plant‐derived compounds—have gained attention for their selective action on cancer cells and low toxicity to normal tissues. Among them, phloridzin, a dihydrochalcone glycoside found primarily in apple trees, shows promising anticancer potential. It modulates multiple signaling pathways involved in cell survival, proliferation, apoptosis, and metastasis. Acting through both intrinsic and extrinsic mechanisms, phloridzin may serve as a complementary or stand‐alone agent in integrative cancer therapy. This review aims to critically examine the current evidence on phloridzin's anticancer effects, underlying mechanisms, bioavailability challenges, and future clinical applications.

## 2 Review Methodology

A comprehensive literature review was conducted to evaluate the anticancer potential of phloridzin, including its pharmacokinetics, mechanisms of action, and therapeutic applications. Using a structured search strategy, relevant scientific articles were retrieved from specialized biomedical databases, including PubMed, Scopus, Google Scholar, Web of Science, and the TRIP database. The search combined Medical Subject Headings (MeSH) terms and Boolean operators (AND, OR, NOT) to refine and optimize the results. The primary MeSH terms and keywords used included “Phloridzin” OR “Phlorizin” OR “Phlorhizin” OR “Phlorizoside” AND (“Cancer” OR “Neoplasms” OR “Carcinogenesis”) AND (“Mechanism of Action” OR “Apoptosis” OR “Cell Cycle Arrest”) AND (“GLUT inhibition” OR “SGLT inhibition”) AND (“Therapeutic Potential” OR “Drug Development”). Additional keyword variations, including “phloridzin derivatives,” “phloridzin bioavailability,” and “phloridzin combination therapy,” were incorporated to capture a broader spectrum of relevant studies. The inclusion criteria for selecting articles were: (1) original research articles, systematic reviews, and meta‐analyses published in peer‐reviewed journals; (2) studies evaluating the pharmacokinetics, bioavailability, and molecular mechanisms of phloridzin in cancer models; (3) studies reporting in vitro, in vivo, or clinical data on the anticancer effects of phloridzin or its derivatives; (4) publications in English. The exclusion criteria included: (1) studies lacking experimental validation of phloridzin's biological activity, (2) non‐peer‐reviewed sources such as preprints, book chapters, and conference abstracts, (3) studies focusing exclusively on the non‐oncological effects of phloridzin (e.g., diabetes, cardiovascular diseases) without relevance to cancer, and (4) redundant or duplicate studies across databases. Following database searches, all retrieved articles were screened by title and abstract, and relevant studies were subjected to a full‐text review. Articles were independently assessed for methodological rigor and relevance, and data extraction was performed to compile information on phloridzin's mechanisms of action, therapeutic potential, and pharmacological limitations. The most important data are summarized in tables and figures.

## 3 General Characteristics of Phloridzin

### 3.1 Natural Sources

In 1835, De Koninck extracted phloridzin from apple tree bark (Londzin et al. 2018). Since then, numerous studies have affirmed apple tree leaves and bark are the chief sources of this compound (Cendrowski et al. 2024; Girotto et al. 2024; Tian et al. 2021). In apple ( *Malus domestica* ) fruits, compared to peeled apples, unpeeled apples are said to be a richer source of phloridzin; the phloridzin content of apple peel is higher as determined to be in the range of 12–418 mg kg^−1^, as compared to apple pulp (4–20 mg kg^−1^) (Dhyani et al. 2018; Ehrenkranz et al. 2005). Furthermore, many studies indicated that old apple cultivars have higher phloridzin amounts than newer ones (Dhyani et al. 2018; Kschonsek et al. 2018). The compound is also found in other plant species, such as sweet potatoes ( *Ipomoea batatas* ), pomegranate ( *Punica granatum* ) fruits, the flesh of Jaeschke's barberry (*Berberis jaeschkeana*), leaves of tea ( *Camellia sinensis* ), strawberries (*Fragaria × ananassa*), chayote ( *Sechium edule* ), Chinese broccoli ( *Brassica oleracea var. alboglabra* ), European plums ( *Prunus domestica* ), lingonberry ( *Vaccinium vitis‐idaea* ), and lettuce ( *Lactuca sativa* ) (Table 1), with a variable distribution (Ehrenkranz et al. 2005; Rana and Bhushan 2016; Tian et al. 2021). Interestingly, some researchers have found that sweet tea contains a hundred times more phloridzin than its commonly assumed source, apples (Shang et al. 2022).

**TABLE 1:** Phloridzin occurrence in different plant species.

Plant species	Plant parts	Solvent	Method	References
*Acca sellowiana*	Mature pulp of fruit	Methanol	In vitro	Verardo et al. (2019)
*Aspalathus linearis*	Plant leaves	Methanol, H_2_O, 1% formic acid	Leaf extract	Stander et al. (2017)
*Berberis jaeschkeana*	Leaves	Methanol	Fruit extract	Belwal et al. (2017)
*Camellia japonica*	Leaves	Water	Leaf extract	Cho et al. (2008)
*Docynia dcne*	Leaves	Acetonitrile, methanol	Leaf extract	Zhang et al. (2021)
*Docynia indica*	Leaves	Methanol, n‐butanol, ethyl acetate, n‐hexane	Leaf extract	Zhang et al. (2018)
*Glycine max*	Seed	Ethanol	Seed extract	Lee et al. (2017)
*Hemerocallis × hybrida*		Ethyl acetate, hexane and methanol	Flower extract	Cichewicz and Nair (2002)
*Fragaria × ananassa*	Flesh of fruit	Acetone	In vitro	Hilt et al. (2003), Khanam et al. (2022)
*Lactuca sativa*	Flesh	Water	Flesh slurry	Altunkaya and Gökmen (2009)
*Lithocarpus polystachyus*	Leaves	Ethanol, methanol	Leaf extract	Chen et al. (2017)
*Lippia graveolens*	Leaf	Methanol	Extract	Lin et al. (2007)
*Nelumbo nucifera*	Seed	Ethanol	Fractions	Ma et al. (2019)
*Pisum sativum*	Seed	Acetone	Extract	Xu et al. (2006)
*Polygonum cuspidatum*	Flower	Methanol	Extract	Sun et al. (2014)
*Prunus persica*	Flesh of fruit	H_2_O, formic acid, methanol	Pulp Extract	Zhang et al. (2019)
*Psidium guajava*	Peel and flesh of the fruit	Formic acid, H_2_O, methanol	Extract	Rojas‐Garbanzo et al. (2017)
*Punica granatum*	Flesh	—	Juice	Poyrazoğlu et al. (2002)
*Pyrus pashia*	Flesh	Ethanol	Pulp	Prakash et al. (2019)
*Rosa canina*	The flesh of the fruit	Methanol, H_2_O, formic acid	Pulp extract	Hvattum (2002)
*Rubus occidentalis*	Flesh	Methanol	Juice	Paudel et al. (2013)
*Solanum lycopersicum*	Flesh	Methanol	Dried powder	Bueno et al. (2018)
*Sechium edule*	Flesh	Methanol	Extract	Aguiñiga‐Sánchez et al. (2017)
*Vaccinium vitis‐idaea*	Flesh	Ethanol, methanol, formic acid	Extract	Bhullar and Rupasinghe (2015)
*Vaccinium macrocarpon*	Flesh	Methanol	Juice	Turner et al. (2005)

### 3.2 Chemical Characterization

**FIGURE 1:**
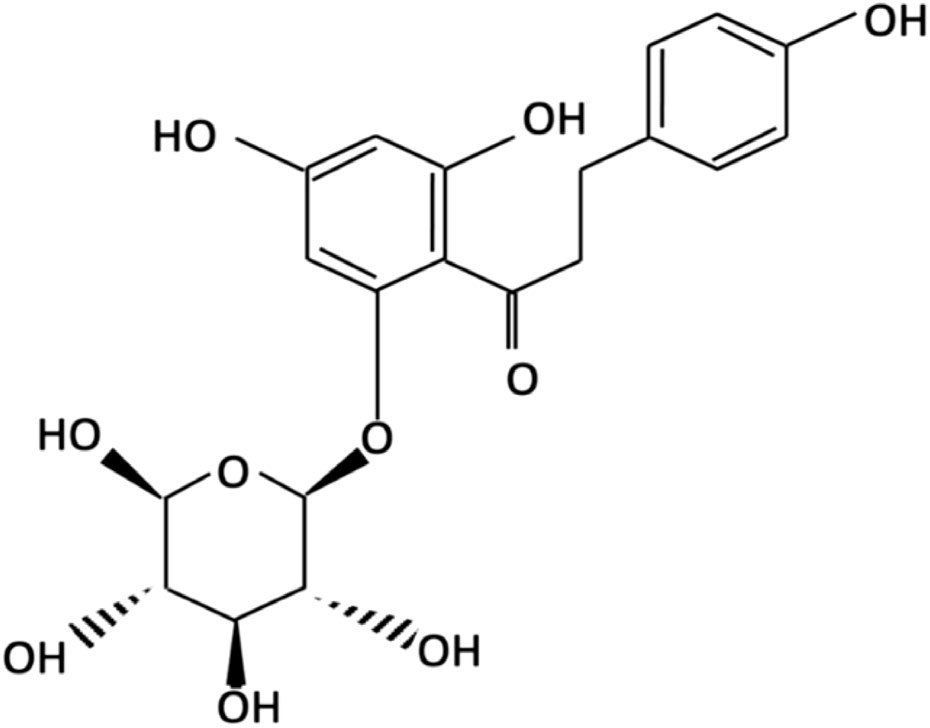
Chemical structure of phloridzin.

Phloridzin belongs to the group of organic compounds and is primarily recognized as a flavonoid O‐glycoside, with a carbohydrate moiety linked to a 2‐phenylchromen‐4‐one flavonoid backbone via an O‐glycosidic bond (Kilit and Aydemir 2022). Phloridzin comprises two aromatic rings connected with a β‐D‐glucopyranose moiety by a C3 chain (Tsao 2010) (Figure 1). The IUPAC name for phloridzin is 1‐[2,4‐dihydroxy‐6‐[(2s,3r,4s,5s,6r)‐3,4,5‐trihydroxy‐6‐(hydroxymethyl)tetrahydropyran‐2‐yl]oxy‐phenyl]‐3‐(4‐hydroxyphenyl)propan‐1‐one with molecular formula C21H24O10 and molecular mass 436.409 g·mol^−1^ (http://pqr.pitt.edu/mol/IOUVKUPGCMBWBT‐QNDFHXLGSA‐N).

**FIGURE 2:**
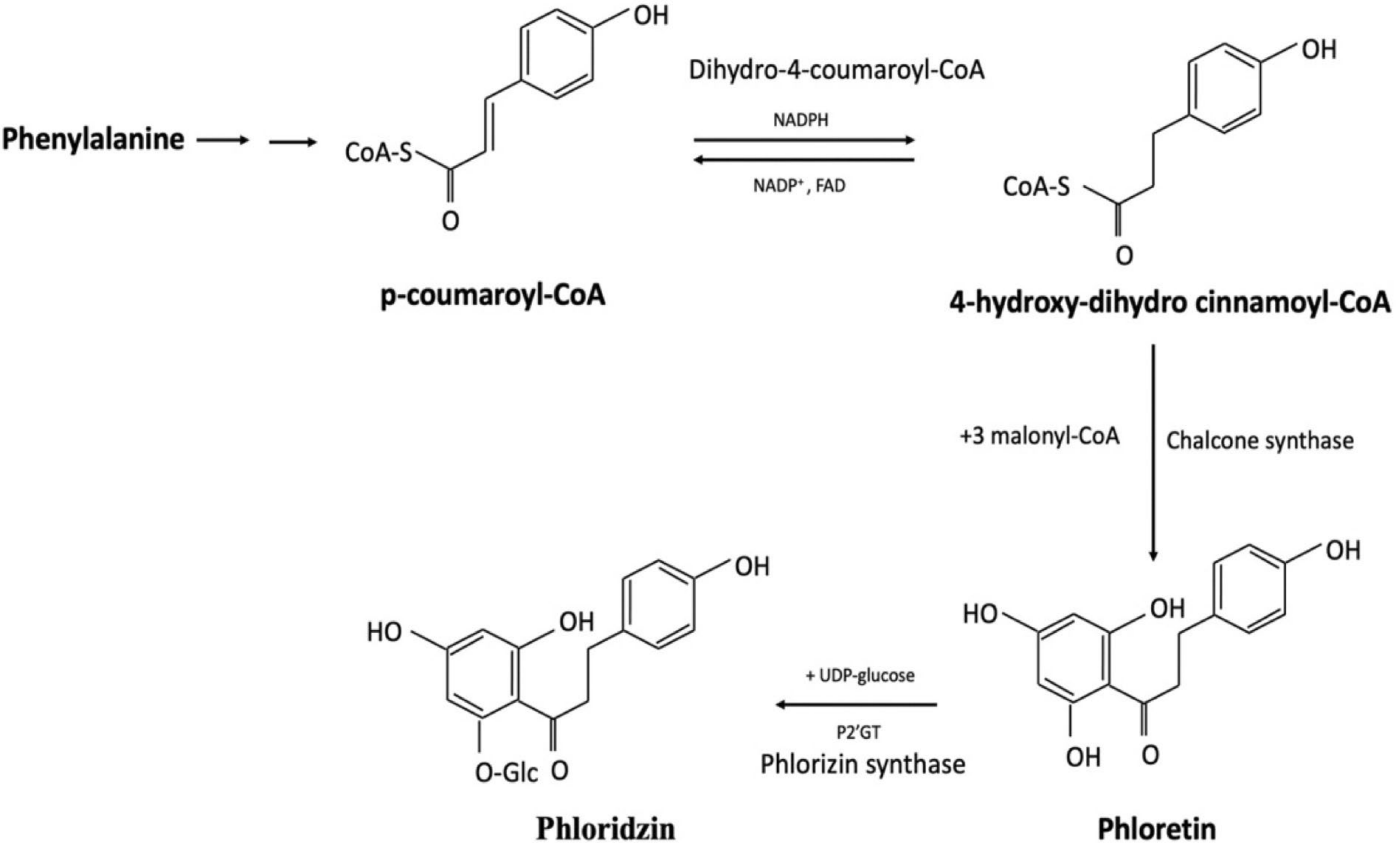
Biosynthetic pathway of phloridzin.

The phloridzin biosynthesis is diverse from common flavonoids, with p‐coumaroyl‐CoA and malonyl‐CoA being the main precursors (Figure 2). The phloridzin synthesis reaction starts with p‐coumaroyl‐CoA generating 4‐hydroxy‐dihydro cinnamoyl‐CoA via the NADPH pathway. The 4‐hydroxy‐dihydro cinnamoyl‐CoA and malonyl‐CoA produce phloretin with chalcone synthase enzymatic action. With the attachment of a glucose moiety to phloretin at position 2′, phlorizin is finally made. This accumulation of a glucose moiety at position 2′ of phloretin indicates that it is the inclusive step in forming phloridzin, with glycosyltransferases (both MdUGT88F1 and its paralog MdUGT88F4) converting phloretin to phloridzin in *Malus* plants (Zhou et al. 2017, 2019). Several studies have indicated that the ENRL‐3 (Enoyl Reductase‐Like genes‐3) and ENRL‐5 contribute to the phloridzin biosynthesis in apples (Dare et al. 2013).

Some researchers investigated the biosynthetic derivatives of phloridzin from the *Malus* species (Table 2). Studies indicate that the quantity and type of phloridzin derivatives in Malus can vary depending on the tissue, variety, developmental stage, sampling time, and pathogen exposure (Łata et al. 2009; Mikulic Petkovsek et al. 2009; Zhang et al. 2007). The derivatives 3‐hydroxy phloretin 20‐*O*‐xyloglucoside and phloretin 20,40‐*O*‐glucoside are only reported in apple juice extracts (Hümmer 2009).

**TABLE 2:** Phloretin derivatives described from *Malus* species.

S. No.	R_1_	R_2_	R_3_	R_4_	Derivatives	References
i.	H	OH	OH	OH	Phloretin	Williams (1961)
ii.	H	OH	OH	Glc	Trilobatin (Phloretin 4´‐*O*‐glucoside)	
iii.	H	OH	Glc	OH	Phloridzin (Phloretin 2´‐*O*‐glucoside)	
iv.	OH	OH	OH	Glc	Sieboldin (3‐Hydroxyphloretin 4´‐*O*‐glucoside)	
v.	OH	OH	Glc	OH	Hydroxyphloridzin	
vi.	OH	OH	OH	OH	3‐Hydroxyphloretin	
vii.	H	OH	O‐Glc‐Xyl	OH	Phloretin 20‐*O*‐xyloglucoside	Williams and Swain (1966)
viii.	H	OH	O‐Glc‐Xyl	OH	Phloretin 20‐*O*‐xylogalactoside	Burda et al. (1990)
ix.	H	O‐coumaroyl	Glc	OH	4‐*O*‐transp‐coumaroyl‐phloridzin	Roemmelt et al. (2003)

Although the majority of *Malus* species contain phloridzin, it is replaced by the derivative trilobatin completely in *Malus trilobata* and complemented by the derivative sieboldin in *Malus sieboldii* and *Malus floribunda* (Williams and Swain 1966). However, investigations on phloridzin and apple scab stated that sieboldin and trilobatin did not occur in similar species (Hunter 1975). Other researchers observed that some offspring of *Malus domestica* crossed with *Malus trilobata* showed both trilobatin and phloridzin (Isayenkova et al. 2006). Additionally, it has been shown that the ortho‐dihydroxyl structure on the B‐ring of sieboldin influences the attachment site of the glucose moiety. Vogt et al. reported that this structural feature affects the sugar attachment site for flavonoid glycosyltransferases (Vogt et al. 1999).

### 3.3 Spectrum of Reported Bioactivities

It is hypothesized that phloridzin contributes to apples' resistance to various diseases, as it extensively exists in apple trees' leaves, bark, and fruit (Ehrenkranz et al. 2005) (Gosch et al. 2010). One of the in vitro studies reported that *Venturia inaequalis* (apple scab) infection in the apple tree is obstructed by aglycone phloretin (Holowczak 1962). Another investigation (Alt and Schmidle 1980) reported the potent impact of phloridzin and phloretin on *Phytophthora cactorum*'s mycelial growth (crown rot of apple) (Roemmelt et al. 2003). An investigation of *Erwinia amylovora* (fire blight bacterium) revealed an antimicrobial effect of phloretin and 4‐O‐cis‐p‐coumaroyl‐phloridzin. Other researchers reported similar observations with phloridzin against *E. amylovora* (Pontais et al. 2008). During co‐incubation with polyphenol oxidase, the research discovered that phloridzin prevents the spore development of *Phlyctaena vagabunda*, a postharvest apple fruit rot (Lattanzio et al. 2001). Several foodstuffs prepared from apples, including juice and extracts, retain an extensive range of biological activities, which might contribute to diseases such as pulmonary dysfunction, cardiovascular diseases, asthma, diabetes, cancer, inflammation, and obesity (Boyer and Liu 2004; Shelke et al. 2024; Wang et al. 2024; Xie et al. 2024). Several laboratory‐based studies (Tables 3 and 4) indicated that phloridzin played a significant role against many vital diseases by showing various actions such as antimicrobial, antioxidant, antiobesity, antiaging, cardioprotective, hypolipidemic, hepatoprotective, immunomodulatory, neuroprotective, anti‐inflammatory, anti‐diabetic, antihypertension, antihyperglycemic, antitumor, etc. (Ni et al. 2024; Sharma et al. 2024; Tripathi and Singh 2024). Phloridzin also displays the potential to counteract malaria, fever, and other infectious diseases (White 2010). A recent study revealed that in mice given a high‐fat diet (HFD), phloridzin significantly lowered the amounts of blood and adipose tissue pro‐inflammatory cytokines and prevented inflammation in the adipose tissue (Tian et al. 2021). Moreover, phloridzin reduced the amounts of pro‐inflammatory cytokines in skin exposed to UVB, thereby reducing acute skin inflammation (Zhai et al. 2015). Similarly, phloridzin acts on cytokine‐driven inflammation and enhances the intestinal anti‐inflammatory response (Zielinska et al. 2019).

**TABLE 3:** Biomedical effects of phloridzin and phloridzin‐enriched extracts.

Biological actions	Plant part used	Plant extract	Bioactive constituent	Type of action	Experimental approach	Results	References
Anti‐inflammatory	Fruit flesh of apple cultivars	Apple extract	Phloridzin	Counteracted Inflammatory disease at the intestinal level	(HPLC‐DAD‐MS/MS) analysis	Ameliorated cytokine‐driven inflammation and exhibited anti‐inflammatory response at the intestinal level	Zielinska et al. (2019)
Antidiabetic			Phloridzin	Regularized hyperglycemia of type 2 diabetes	Phloridzin in sterile saline (20 mg kg^−1^ body weight) daily	Hyperglycaemia stabilization in type 2 diabetic mice by reducing lipopolysaccharide in serum and alteration of gut microbiota	Mei et al. (2016)
Antihypertension			Phloridzin	Reduced diabetes	Phloridzin (0.4 g/kg body wt. per day) mixed in 20% propylene glycol the solution is given for 4 weeks	In diabetic rats, hypertension induction and SGLT2 activity were inhibited.	Osorio et al. (2010)
Antihyperglycemic	Leaves of *Malus hupehensis*	Extract	Phloridzin	Showed inhibitory effects on glucosidase	Elution was isolated through preparative HPLC, using acetonitrile (30%) and acetic acid (0.1%)	Exhibited concentration‐reliant inhibitory effects on glucosidase	Lv et al. (2019)
Antitumor	leaves of *Malus crab apples*	Extract	Phloridzin	Inhibited cancer cell growth	Isolated from *Malus crab‐apple* leaves through HPLC	Phloridzin isolated from Crab apple leaves showed an antitumor effect via cancer cell growth inhibition.	Qin et al. (2015)
Antimicrobial	Leaves	Extract	Phloridzin	Exhibited antimicrobial activity	*Malus domestica* leaf extract tested through HPLC	Ethyl acetate extract of leaves tested against *Candida glabrata, faecalis, Staphylococcus aureus, Enterococcus* strains	Sowa et al. (2016)
Anti‐obesity			Phloridzin	Reduced the excessive fat in diet‐induced obese mice	A higher dose of the fat diet with phloridzin was given to male mice for 16 weeks	A higher amount of phloridzin augmented the resistance to insulin and obesity in excessive fat diet‐induced overweight mice	Shin et al. (2016)
Anti‐aging	Branches of dwarf apple		Phloridzin	Enhancement of superoxide dismutase (SOD) and SIRT1 action	Comparison of 1H and 13C NMR spectra	The anti‐aging effect observed in yeast by the escalation in superoxide dismutase (SOD) and SIRT1 activity	Xiang et al. (2011)
Anti‐oxidant	Leaves	Crude extract	Phloridzin	Apoptosis of H_2_O_2_‐induced HepG2 cells were reduced	Ultra‐high‐performance liquid chromatography	Phloridzin significantly prevented oxidative stress	Fan et al. (2020)
Hypolipidemic			Phloridzin	Counteracted hepatic injury in type 2 diabetic mice	Intragastric administration of phloridzin (98%) along with standard saline solution for 10 weeks	Counteracted hepatic injury in type 2 diabetic mice by the reduction of body weight, glucose level in the blood, total cholesterol and triglycerides	Lu et al. (2012)
Hepatoprotective	Phloridzin	Oral administration	Phloridzin	Counteracted oxidative stress and inflammation	Oral administration of phloridzin (40 mg kg^−1^ day^−1^) for ten successive days	Phloridzin prevented hepatic injury in rats by countering oxidative stress, apoptosis, as well as inflammation in the hepatic tissues of rats	Khalifa et al. (2017)
Neuroprotective	Phloridzin			Influenced memory storage	Before use, phloridzin (3‐300 g/kg) dissolved in saline and given intraperitoneally	Phloridzin improved memory storage but failed to recover memory loss	Boccia et al. (1999)
Immunomodulatory	Fruits of *Malus baccata*	Extracts		Radioprotective and immunomodulatory action	Fruit extract of *Malus baccata* contained phloridzin (18.24%)	Phloridzin isolated from *Malus baccata* exhibited radioprotective as well as immunomodulatory action	Wang et al. (2016)
Cardioprotective			Phloridzin		Administration of phloridzin (98%) intragastrically (8 to 18 week) excluding hypoglycemic therapy	Phloridzin counteracted the expansion of diabetic cardiomyopathy by regulating the expression of crucial proteins	Cai et al. (2013)

**TABLE 4:** Occurrence and mode of administration of phloridzin originating from different plant species.

Plant species	Plant parts	Preparation/extraction form	Mode of administration	Action	Country	References
*Malus domestica*	Fruit peel	Ethanolic extract	In vitro	Inhibition of LDL‐cholesterol oxidation	Canada	Thilakarathna et al. (2013)
*Malus domestica*	Leaves	Ethanolic extract	In vitro	Cancer	Portugal	Roleira et al. (2015)
*Lithocarpus polystachyus*	Leaves	Ethanolic extract	In vitro	Maintain blood glucose level	China	Sun et al. (2015)
*Lithocarpus polystachyus*	Leaves	Ethanolic extract		Anti‐hyperlipidemic and anti‐hyperglycemic action	China	Zhang et al. (2021)
*Malus domestica*	Leaves	Ethyl acetate, water and methanolic extract	In vitro	Antimicrobial and anti‐oxidant action	Poland	Sowa et al. (2016)
*Eleutherococcus senticosus*	Plant root	Methanolic and aqueous extract	In vitro	Epidermal basal cell proliferation stimulation	Republic of Korea	Choi et al. (2016)
*Malus domestica*	Fruit peel	—	In vivo	Anti‐oxidant and anti‐aging activity	China	Wang et al. (2019)
*Malus hupehensis*	Plant leaves	Ethanolic extract	In vitro	Anti‐diabetic action	China	Lv et al. (2019)
*Lithocarpus polystachyus*	Plant leaves	—	In vivo	Anti‐hyperglycemic and anti‐hyperlipidemic action	China	Zhang et al. (2021)

## 4 Mechanism of Anti‐Tumor Action of Phloridzin

Cancer cells exhibit altered nutrient uptake, particularly an increased dependency on glucose metabolism, to sustain their rapid proliferation, survival, and metastatic potential. This metabolic shift, known as the Warburg effect, rewires key oncogenic pathways, enhances glycolytic flux, and promotes immune evasion (Li et al. 2024; Papaneophytou 2024). Natural compounds, including flavonoids and dihydrochalcones, have demonstrated potent anticancer effects by targeting metabolic vulnerabilities, inducing apoptosis, and disrupting key signaling pathways (Dias et al. 2021). Among these bioactive compounds, phloridzin has emerged as a promising anticancer agent due to its unique ability to inhibit glucose transporters (GLUTs), suppress JAK2/STAT3 signaling, modulate apoptosis, and interfere with cell cycle regulation (Jia et al. 2021; Ni et al. 2024).

### 4.1 Metabolic Disruption via GLUT Inhibition

The cytotoxicity of phloridzin stems from its ability to inhibit GLUT1 and GLUT2, restricting glucose transport in cancer cells and thereby impairing their energy metabolism and biosynthetic capacity, ultimately suppressing tumor growth (Kilit and Aydemir 2022; Wu et al. 2009). GLUT1 is overexpressed in many aggressive cancer types (e.g., breast, lung, colon), supporting enhanced glycolysis and tumor progression. Phloridzin competitively inhibits GLUT1, leading to reduced glucose uptake, ATP depletion, and metabolic stress‐induced apoptosis (Wu et al. 2009). Phloridzin exhibits high specificity for GLUT2, particularly in triple‐negative breast cancer (TNBC), where it blocks glucose transport, disrupts energy metabolism, and reduces tumor growth (Wu et al. 2018). By limiting glucose metabolism, phloridzin shifts energy production towards oxidative phosphorylation (OXPHOS), leading to an increase in reactive oxygen species (ROS) and mitochondrial‐mediated apoptosis (Brockmueller et al. 2021). Recent studies suggest that phloridzin enhances the efficacy of metabolic inhibitors such as 2‐deoxyglucose (2‐DG) by synergistically inhibiting glycolytic flux, further sensitizing cancer cells to metabolic stress (Zhang et al. 2021).

### 4.2 Apoptosis Induction

**FIGURE 3:**
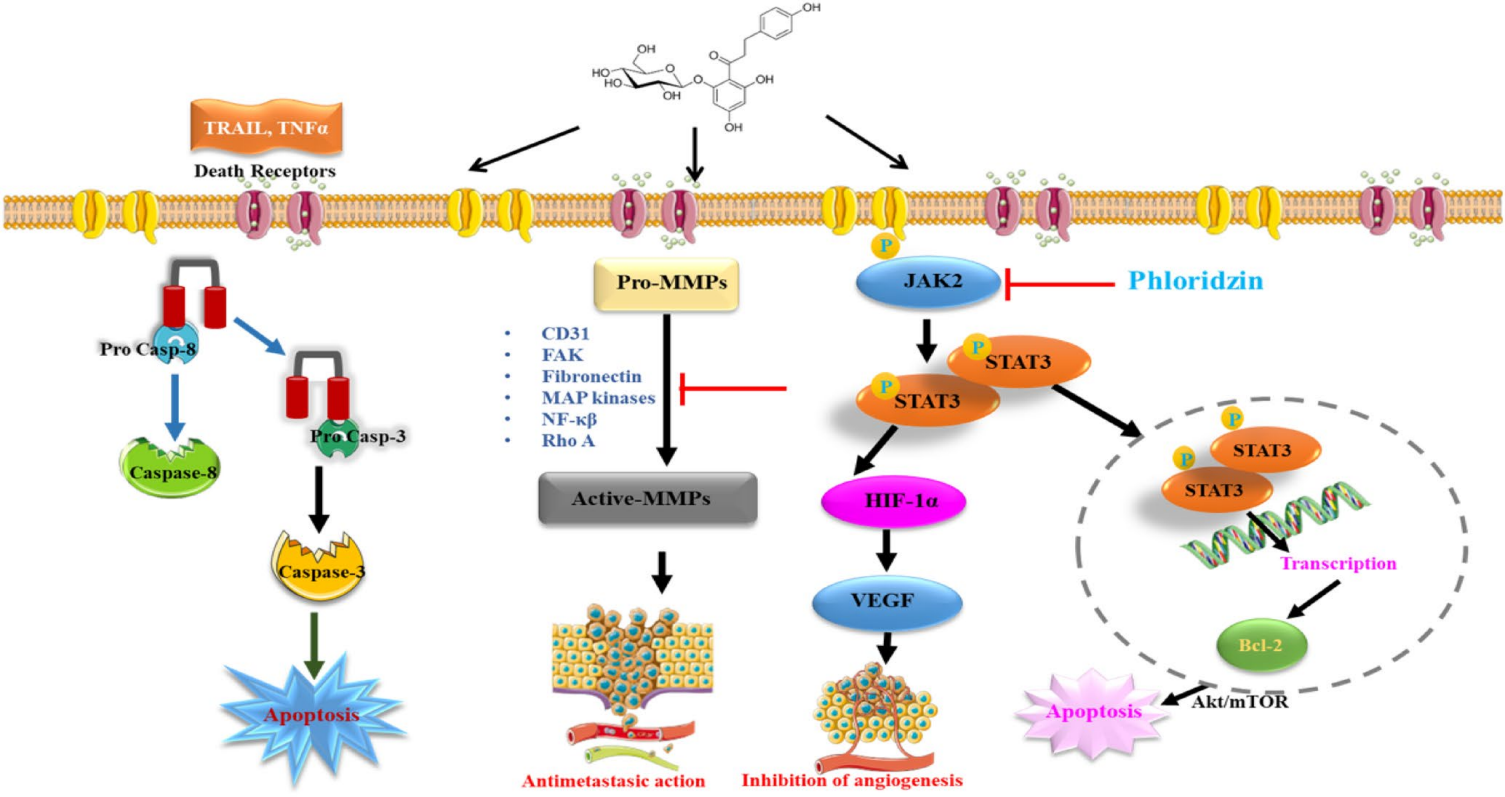
Molecular mechanisms of phloridzin's antitumor activity, highlighting its role in apoptosis induction, metastasis suppression, and angiogenesis inhibition. Phloridzin inhibits JAK2/STAT3 signaling, preventing STAT3 phosphorylation and nuclear translocation, which downregulates Bcl‐2 expression and promotes apoptosis via the Akt/mTOR pathway. Additionally, phloridzin suppresses HIF‐1α‐mediated VEGF expression, thereby inhibiting tumor angiogenesis. The compound also inhibits pro‐matrix metalloproteinases (pro‐MMPs) activation, reducing the expression of key metastatic proteins (CD31, FAK, Fibronectin, MAP kinases, NF‐κB, and Rho A), leading to antimetastatic effects. Moreover, phloridzin enhances TRAIL/TNFα‐induced apoptosis, activating the caspase‐8 and caspase‐3 cascade. Akt, Protein kinase B; Bcl‐2, Anti‐apoptotic protein; CD31, Endothelial adhesion molecule; Casp‐3, Executioner caspase in apoptosis; Casp‐8, Initiator caspase in apoptosis; FAK, Focal adhesion kinase; Fibronectin, Cell adhesion glycoprotein; GLUT, Glucose transporter; HIF‐1α, Hypoxia‐inducible factor‐1 alpha; JAK2, Janus kinase 2; MAPK, Mitogen‐activated protein kinase; MMPs, Matrix metalloproteinases; NF‐κB, Nuclear factor kappa B; P‐Phlor, Phloridzin; Pro‐Casp‐3, Inactive caspase‐3 precursor; Pro‐Casp‐8, Inactive caspase‐8 precursor; Pro‐MMPs, Inactive metalloproteinases; Rho A, GTPase regulating migration; STAT3, Signal transducer and activator of transcription 3; TNFα, Tumor necrosis factor‐alpha; TRAIL, TNF‐related apoptosis‐inducing ligand; VEGF, Vascular endothelial growth factor.

Phloridzin triggers apoptosis through the intrinsic (mitochondrial) and extrinsic (death receptor) pathways, as demonstrated in leukemia, colorectal, and liver cancer models (Arumuggam et al. 2017; Jin et al. 2020) (Figure 3). Phloridzin upregulates caspase‐3 and caspase‐9 expression, initiating apoptotic cascades in non‐small cell lung cancer (NSCLC) and colorectal cancer cells (Min et al. 2015) (Kim et al. 2022). The compound downregulates Bcl‐2 (anti‐apoptotic) while upregulating Bax (pro‐apoptotic), resulting in cytochrome c release and activation of the apoptotic machinery (Sandhya VG Nair and Rupasinghe 2014; Wu et al. 2009). Phloridzin enhances tumor necrosis factor‐related apoptosis‐inducing ligand (TRAIL)‐induced apoptosis, increasing death receptor (DR4/DR5) expression and sensitizing cancer cells to apoptotic signals (Kim et al. 2022). A recent study highlighted that phloridzin triggered apoptosis in oesophageal cancer cell lines and inhibited cell autophagy. Thereby, phloridzin repressed the progression of oesophageal cancer by acting as an antagonist to the JAK2/STAT3 signaling pathway (Jia et al. 2021). Fatty acid ester derivatives of phloridzin are also considered potential chemotherapeutic agents. Their anti‐tumor effects are mediated by the downregulation of several key proteins involved directly or indirectly in cell cycle regulation, modulation of DNA topoisomerase IIα activity, and epigenetic processes, ultimately leading to cell cycle arrest and apoptosis (Nair and Rupasinghe 2014). The anti‐tumor action of an ester of phloridzin is allied to the down‐regulation of the anti‐apoptotic gene (BCL2), several growth factor receptors (EBFR family, IGF1R/IGF2, PDGFR), their signaling associates (Ras/Raf/MAPK, PI3k/AKT/mTOR), and the machinery of the cell cycle in addition to epigenetic regulators (Nair and Rupasinghe 2014). The anti‐tumor effects of phloridzin, acylated with docosahexaenoic acid, on Jurkat cells, were linked to activation of caspase activity, DNA fragmentation, and STAT3 phosphorylation selective down‐regulation (Arumuggam et al. 2017). To sum up, phloridzin triggers the death of cancer cells via either direct or indirect pathways. Notably, the group of dihydrochalcones, to which phloridzin belongs, are one of the promising anti‐cancer agent classes as they were shown to induce selective cell death in carcinoma cells without upsetting normal cells (Salehi et al. 2021). Dihydrochalcones cause apoptotic cell death via TNF superfamily members, predominantly tumor necrosis factor‐related apoptosis‐inducing ligand (TRAIL) through the interface with death receptors, i.e., DR4 orR5 in numerous cancer cells without a harmful effect on normal tissue. The commencement of cellular apoptotic processes and the anti‐proliferative activity, closely associated with cell cycle arrest, are two important regulatory elements and are essential steps in cell cycle development. Deregulation of cell cycle processes results from changes in the expression or post‐transcriptional alterations of cyclins and cyclin‐dependent kinases by dihydrochalcones (Orlikova et al. 2011).

### 4.3 Inhibition of Oncogenic JAK2/STAT3 Signaling

The JAK2/STAT3 pathway is an important driver of tumor progression, immune evasion, and therapy resistance (Figure 3); persistent STAT3 activation is linked to poor prognosis in multiple malignancies, including esophageal, breast, and colorectal cancer (Jia et al. 2021). Phloridzin suppresses JAK2 phosphorylation, preventing STAT3 activation and reducing downstream expression of key oncogenes such as Bcl‐xL (anti‐apoptotic), Cyclin D1 (cell cycle regulator), and VEGF (angiogenesis promoter) (Jia et al. 2021).

### 4.4 Suppression of PI3K/AKT/mTOR and MAPK/ERK Pathways

The phosphoinositide 3‐kinase (PI3K)/protein kinase B (AKT)/mechanistic target of rapamycin (mTOR) pathway and the mitogen‐activated protein kinase (MAPK)/extracellular signal‐regulated kinase (ERK) pathway are key oncogenic signaling networks that regulate cell proliferation, survival, and metastasis (Du et al. 2016). Their dysregulation contributes to tumor progression, resistance to apoptosis, and therapeutic resistance, making them critical targets in cancer treatment strategies. Phloridzin effectively inhibits these pathways, reducing tumor growth and overcoming drug resistance (Kang et al. 2020). In prostate cancer models, phloridzin reduces AKT phosphorylation, leading to downregulation of Sp1 and Sp3/4 transcription factors, which regulate cancer cell survival and chemoresistance (Kang et al. 2020). In glioblastoma and lung cancer models, phloridzin inhibits ERK1/2 phosphorylation, leading to cell cycle arrest in the G0/G1 phase and impaired proliferation (Hou et al. 2025; Min et al. 2015). Recent in vivo studies also highlight that phloridzin, when combined with mTOR inhibitors (e.g., rapamycin), synergistically enhances tumor regression in a colorectal cancer xenograft model (M. Chen et al. 2021).

### 4.5 Anti‐Metastatic and Anti‐Angiogenic Effects

Metastasis is the leading cause of cancer‐related mortality (Lusby et al. 2025). Phloridzin disrupts multiple steps in the metastatic cascade, including tumor cell invasion, migration, and angiogenesis. Phloridzin downregulates MMP‐2 and MMP‐9, enzymes essential for extracellular matrix degradation, preventing cancer cell dissemination (Hsiao et al. 2019). By inhibiting VEGF expression, phloridzin restricts tumor vascularization, reducing nutrient supply and impeding metastatic spread (Jia et al. 2021).

### 4.6 Scientific Studies on the Anti‐Cancer Properties of Phloridzin

Many studies demonstrate important anti‐tumor properties of phloretin, explaining the antagonistic action on tumor cells in different preclinical in vitro models of breast cancer, cervical cancer, colon cancer, lung cancer, gastric cancer, oral cancer, oesophageal cancer, and liver cancer and prostate cancer (Abouelenein et al. 2023; de Freitas Rodrigues et al. 2023; Fernando et al. 2023; Jang et al. 2024) (Table 5). Many in vitro studies on this promising flavonoid have been compiled in this part, comprising its considerable anti‐cancer potential as an anti‐angiogenic, anti‐proliferative, and anti‐cancer compound. Attention was also given to the molecular mechanism of action of phloretin within this context.

**TABLE 5:** In vitro and in vivo studies on the anti‐cancer effects of phloridzin.

Types of cancer	Cell line model/animal model	Mechanisms and actions involved	References
**In vitro studies**			
Non‐Small Cell Lung Carcinoma (NSCLC)	A549, H1299, Beas‐2b	**↑** Caspase‐3, −9, **↑** PARP, **↓** Bcl‐2 (dose‐dependent) **→** induction of apoptosis. **↑** P38 MAPK and JNK1/2 activation **→** increased apoptotic signaling.	Min et al. (2015)
Breast Cancer (TNBC)	MDA‐MB‐231, MCF‐10A	**↓** GLUT2 expression **→ ↓** glucose uptake and ATP production **→** energy deprivation and metabolic stress. **↓** cell proliferation and migration, **↑** cell cycle arrest (p53‐dependent).	Wu et al. (2018)
Cervical Cancer	SiHa	**↓** MMP‐2, ↓MMP‐3, ↓cathepsin S expression **→ ↓** extracellular matrix degradation **→ ↓** invasion, **↓** migration, **↓** angiogenesis.	q
Prostate Cancer	LNCaP, CWR22Rv1, PC‐3, DU145, WPMY‐1	**↓** PI3K/AKT and MEK/ERK1/2 phosphorylation **→ ↓** EGFR activation **→ ↓** Sp1, VEGF, Survivin expression **→** cell cycle arrest and apoptosis.	Kang et al. (2020)
Colon Cancer	DLD1, HCT116	**↑** TRAIL‐induced apoptosis **→ ↑** cleaved PARP, caspase‐3, −8, −9 activation **→** programmed cell death and DNA fragmentation.	Kim et al. (2022)
Glioblastoma	U87	**↓** PI3K/AKT/mTOR signaling **→ ↓** tumor cell survival **→ ↑** temozolomide (TMZ) sensitivity and reduced chemoresistance.	Hou et al. (2025)
Leukemia (T‐ALL Model)	Jurkat, K562	**↑** Caspase‐3 activation **→ ↓** STAT3 phosphorylation **→ ↑** DNA fragmentation and apoptosis.	Arumuggam et al. (2017)
**In vivo studies**			
Triple‐Negative Breast Cancer (TNBC)	BALB/c nude mice	**↓** Tumor growth by **↓** paxillin/FAK, Src, α‐SMA expression. **↑** E‐cadherin, p53, and p21 levels **→** suppression of epithelial‐mesenchymal transition (EMT).	Wu et al. (2018)
Cervical Cancer	Nude mice	**↓** MMP‐2, ↓MMP‐3, ↓cathepsin S levels **→ ↓** fibronectin, vimentin, Rho A expression **→** EMT reversal, reduced invasiveness, and tumor regression.	Hsiao et al. (2019)
Colorectal Cancer	BALB/c nude mice	**↑** Bax pro‐apoptotic protein **→ ↓** Bcl‐2, NF‐κB, and MMP‐9 **→** apoptosis induction and tumor growth inhibition.	Jin et al. (2020)
Liver Cancer	HepG2 mice model	**↓** Akt/mTOR pathway activation **→ ↑** mitochondrial dysfunction, **↑** oxidative stress **→** intrinsic & extrinsic apoptosis activation.	Wu et al. (2009)
Glioblastoma	BALB/c nude mice	**↓** PI3K/AKT/mTOR signaling **→ ↓** tumor volume, **↑** apoptosis rate, **↑** sensitivity to temozolomide treatment.	Hou et al. (2025)

Abbreviations: ↑, Upregulation or increase; ↓, Downregulation or decrease; Akt, Protein kinase B; Bax, Pro‐apoptotic protein; Bcl‐2, Anti‐apoptotic protein; Casp‐3, Caspase‐3; Casp‐8, Caspase‐8; Casp‐9, Caspase‐9; EGFR, Epidermal growth factor receptor; EMT, Epithelial‐mesenchymal transition; FAK, Focal adhesion kinase; GLUT2, Glucose transporter 2; JNK, c‐Jun N‐terminal kinase; MAPK, Mitogen‐activated protein kinase; MMP‐2, Matrix metalloproteinase‐2; MMP‐3, Matrix metalloproteinase‐3; MMP‐9, Matrix metalloproteinase‐9; NF‐κB, Nuclear factor kappa B; PARP, Poly (ADP‐ribose) polymerase; PI3K, Phosphatidylinositol 3‐kinase; Rho A, Ras homolog family member A; Src, Proto‐oncogene tyrosine‐protein kinase Src; STAT3, Signal transducer and activator of transcription 3; TMZ, Temozolomid; TNBC, Triple‐negative breast cancer; TRAIL, TNF‐related apoptosis‐inducing ligand; VEGF, Vascular endothelial growth factor; α‐SMA, Alpha smooth muscle actin.

Phloridzin from sweet tea prevented cell proliferation, migration, and invasion in oesophagal cancer cells. Phloridzin triggered apoptosis and antagonized autophagy in the tumor cells. Notably, the anti‐tumor activity of phloridzin was due to the Pleiotropic suppression of proteins such as P62/SQSTM1 and LC3 І/II in the JAK/STAT signaling pathway, which is responsible for oesophagal cancer growth, metastasis, and apoptosis, thus affecting the overall development of cancer cells. Further bioinformatics studies revealed that phlorizin might be involved in pleiotropic effects, such as the ‘JAK/STAT signaling pathway’ (hsa04630), ‘MAPK signaling pathway’ (hsa04010) and ‘apoptosis’ (hsa04210). Therefore, phlorizin suppressed oesophagal cancer development by antagonizing the JAK2/STAT3 signaling pathway (Jia et al. 2021). It has an anti‐cancer effect on acute lymphoblastic leukemia (ALL) as a type of blood cancer (Arumuggam et al. 2017). The study revealed that phloridzin and DHA, in combination, had a superior cytotoxic effect on Jurkat T‐ acute lymphoblastic leukemia (T‐ALL) cells compared to K562 CML cells. The In vivo studies revealed that Phloridzin and DHA also pointedly decreased the cell viability in Jurkat and K562 cells in a dose‐dependent manner, blocked cell proliferation, and selectively triggered apoptosis. Furthermore, the Phloridzin and DHA or alone, declined cell viability and ATP levels, improved intracellular LDH release, and caused extensive morphological alterations in both Jurkat and K562 cells. Compared to normal cells, PZ‐DHA affected cell proliferation and apoptosis in Jurkat and K562 cells by causing caspase activation, DNA fragmentation, and selective down‐regulation of STAT3 phosphorylation. Phloretin exhibits anti‐metastatic properties due to its glucose transporter (GLUT) inhibitory activity and anti‐cancer effect on human liver cancer cells (C. H. Wu et al. 2009). The authors evaluated virtual molecular channels and pathways mechanizing their cytotoxic effects on HepG2 cells. PZ‐induced cytotoxicity is correlated with the expression of GLUT2. Glucose treatment showed reverse apoptosis in HepG2, which was not reversed in GLUT2 siRNA knockdown‐induced HepG2 apoptosis. According to Western blot analysis, Akt and Bcl‐2 family signaling pathways were responsible for PZ‐induced cell death in HepG2 cells. The results suggest that PZ‐induced apoptosis in HepG2 cells involves inhibiting GLUT2 glucose transport mechanisms. The phloridzin fatty acid esters also blocked the progression of leukemia and carcinoma cells, whereas human or rat‐derived hepatocytes remained unaffected (Nair and Rupasinghe 2014). Here, the mechanism of action involves the inhibition of DNA topoisomerases IIa activity, which triggered the G0/G1 phase's blockage, apoptosis via caspase‐3 activation, reduced ATP concentration, and interrupted membrane potential in HepG2 cells. Different research evaluated the effects of apple dihydrochalcone and phloretin on breast cancer cell viability in in vitro conditions (M. Chen et al. 2021). The probable mechanism involved effective suppression of glucose‐starvation‐ and chemotherapeutic‐induced cytoprotective autophagy in breast cancer cell lines estrogen‐receptor‐positive MCF7 and triple‐negative MDA‐MB‐231 cells likely through downregulation of mTOR/ULK1 signaling after phloretin treatment. Further in vitro and in vivo studies (Fernando et al. 2016) showed that fatty acid ester derivatives, i.e., phloridzin–DHA, were selectively toxic to breast cancer cells. The cytotoxicity was tested on mammary carcinoma cells (MDA‐MB‐231, MDA‐MB‐468, 4 T1, MCF‐7, and T‐47D). Results revealed that phloridzin–DHA showed anti‐proliferative activity in MDA‐MB‐231 cells and arrested cell division at the G2/M phase when administered at a sub‐cytotoxic concentration. Phloridzin–DHA triggered cell apoptosis and down‐regulated cyclin B1 and cyclin‐dependent kinase 1 expression. Phloridzin–DHA also suppressed MDA‐MB‐231 xenograft growth in vivo in immune‐deficient mice. One another study reported the cytotoxic activity of phloridzin on the MCF‐7 breast cancer cell line in a dose‐dependent manner and selective cytotoxicity at its low concentration in cancer cell lines such as 22RV1, U87 and A54. Still, there was no significant cytotoxicity on the MDA‐MB‐231 cell line, and the mechanism involved the anti‐tumor potential of phloridzin due to the glucose transmembrane transport inhibition (Kilit and Aydemir 2022). In breast cancer cells, including estrogen‐receptor‐positive (MCF7) and triple‐negative (MDA‐MB‐231), phloretin was shown to downregulate autophagy‐coupled genes (Roy et al. 2022). Low‐glucose and glucose‐deficient media were found to be responsible for autophagosomal marker LC3B‐II manifestation inhibition, which further underscores the blocked glucose‐starvation‐induced cytoprotective autophagy. Along this line, chemotherapy treatment of ruthenium and phloretin intricated enhanced cell death in breast carcinoma conditions and promoted Bcl2 and Bax by interrupting PI3K/Akt/mTOR signaling. Phlorizin's application as a therapeutic drug may be limited due to low water solubility and minimum absorption capability (Gu et al. 2022). To strengthen its application, a study investigated a nanostructured lipid carrier (NLC) loaded with this flavonoid where a transmission electron microscopy reflected its spherical structure, a sustained release fashion of phlorizin and improved absorption capability in vitro, which suggests that the encapsulation technique has a significant role in enhancing its oral absorption and bioavailability.

## 5 Combination Therapies and Enhanced Anti‐Tumor Effects

The combination of natural bioactive compounds with standard cancer therapies has gained interest due to their ability to enhance efficacy and reduce toxicity. Phloridzin, a dihydrochalcone with glucose transporter (GLUT) inhibitory properties, has demonstrated significant anticancer potential when used in combination with chemotherapy and other natural compounds. In a study by Hou et al., phloridzin demonstrated potent anticancer effects in glioblastoma, particularly when combined with temozolomide (TMZ) (Hou et al. 2025). Phloridzin, a GLUT inhibitor, disrupts glucose metabolism, limiting glioblastoma cell proliferation and migration. It also inhibits epithelial‐mesenchymal transition (EMT) by downregulating N‐cadherin and upregulating E‐cadherin, thereby reducing tumor invasiveness. Additionally, phloridzin targets the PI3K/AKT/mTOR pathway, a key driver of glioblastoma progression, by suppressing PI3K, p‐AKT, and p‐mTOR expression, ultimately decreasing tumor cell survival. When combined with TMZ, phloridzin enhances cytotoxicity, lowers TMZ resistance, and reduces the required TMZ dosage, potentially minimizing treatment toxicity. These findings suggest phloridzin as a promising adjunct therapy that improves glioblastoma treatment outcomes by targeting metabolism, EMT, and survival pathways while sensitizing tumors to chemotherapy (Hou et al. 2025). A study conducted by Sarimahmut et al. investigated its ability to counteract DNA damage induced by the chemotherapeutic agent mitomycin C in human lymphocytes (Sarimahmut et al. 2022). Using chromosome aberration, micronucleus, and comet assays, the study demonstrated that phloridzin effectively reduced mitomycin C‐induced genotoxicity at concentrations ranging from 125 to 500 μg/mL, with statistically significant results (*p* < 0.05). Additionally, the combination of phloridzin with mitomycin C led to a marked decrease in the mitotic index, suggesting an inhibitory effect on cell division. Interestingly, gender did not appear to influence the antigenotoxic or antiproliferative effects observed in the study (Sarimahmut et al. 2022). These findings indicate that while phloridzin exhibits protective properties against DNA damage, it may also interfere with the efficacy of genotoxic‐based chemotherapeutic agents, highlighting the need for further research on its potential interactions with cancer treatments.

## 6 Therapeutic Perspectives, Limitations and Clinical Gaps of Phloridzin in Oncology

Phloridzin has demonstrated significant anticancer potential in preclinical studies, exhibiting pro‐apoptotic, anti‐proliferative, anti‐metastatic, and metabolic regulatory effects across multiple cancer models. Its primary mechanisms include glucose transporter inhibition (GLUT1, GLUT2), suppression of PI3K/AKT/mTOR signaling, and modulation of apoptotic pathways (González‐Gallego et al. 2010; Qin et al. 2015). Additionally, phloridzin limits TNF‐α mRNA and IL‐8 expression, thereby suppressing CXCL10 production in LPS‐stimulated human acute monocytic leukemia cell lines (Jung et al. 2009). Dietary phenolic compounds, including phloridzin, have been found to reduce cancer risk and alleviate drug resistance, supporting their potential as adjunct therapies (Fernando et al. 2023, 2016). Despite its potent anticancer effects, phloridzin's clinical application is hindered by its poor bioavailability. It is rapidly hydrolyzed by intestinal β‐glucosidases into phloretin, which, although more bioavailable, has distinct pharmacokinetic and pharmacodynamic properties, limiting its systemic availability (Crespy et al. 2001). To address this, regioselective enzymatic acylation has been used to synthesize fatty acid esters of phloridzin, incorporating long‐chain saturated, mono‐, and polyunsaturated fatty acids. These derivatives showed increased cytotoxic potency against hepatocellular carcinoma, breast adenocarcinoma, and leukemia cells compared to the parent compound (Cardile et al. 2005). Among these modifications, phloridzin‐docosahexaenoate (PZ‐DHA), synthesized through lipase B enzyme‐catalyzed acylation, exhibited superior cellular penetration and enhanced cytotoxic effects on malignant cells (Bhullar et al. 2013; Khalid et al. 2018). Conjugation with DHA improved pharmacokinetics and therapeutic index, making PZ‐DHA a strong chemotherapeutic candidate. Importantly, phloridzin's anticancer activity is not limited to glucose uptake inhibition. It also influences oncogenic cascades such as PI3K/AKT/mTOR and JAK2/STAT3, which are central to tumor development. By modulating these pathways, phloridzin disrupts cell survival signals and facilitates apoptosis, particularly via mitochondrial dysfunction and caspase activation. Furthermore, esterified derivatives of phloridzin have shown potential in inhibiting DNA topoisomerase IIα, inducing cell cycle arrest, and affecting epigenetic regulators in hepatocellular carcinoma models. Conjugation with DHA improved pharmacokinetics and therapeutic index, making PZ‐DHA a strong chemotherapeutic candidate. Importantly, phloridzin's anticancer activity is not limited to glucose uptake inhibition. It also influences oncogenic cascades such as PI3K/AKT/mTOR and JAK2/STAT3, which are central to tumor development. By modulating these pathways, phloridzin disrupts cell survival signals and facilitates apoptosis, particularly via mitochondrial dysfunction and caspase activation. Furthermore, esterified derivatives of phloridzin have shown potential in inhibiting DNA topoisomerase IIα, inducing cell cycle arrest, and affecting epigenetic regulators in hepatocellular carcinoma models (Nair and Rupasinghe 2014). The chemopreventive role of phloridzin in hepatocellular carcinoma (HCC) is particularly noteworthy, as HCC remains a major cause of cancer‐related mortality (Stagos et al. 2012). Research indicates that phloridzin and its derivatives can suppress hepatocarcinogenesis, particularly in liver cirrhosis and aflatoxin‐induced hepatic malignancies (Naghibi et al. 2013), (Lemma et al. 2017). Current therapeutic options for HCC, including surgical resection, liver transplantation, transarterial embolization, and oral multikinase inhibitors, have shown limited survival benefits (Niederberger et al. 2020). Thus, incorporating phloridzin‐based strategies into existing HCC treatments could enhance efficacy and patient outcomes. Emerging evidence suggests that phloridzin derivatives enhance the efficacy of conventional chemotherapies. In glioblastoma models, phloridzin improves the cytotoxic effects of temozolomide (TMZ) by suppressing PI3K/AKT/mTOR signaling, leading to increased apoptosis (Hou et al. 2025). Additionally, PZ‐DHA has been shown to potentiate paclitaxel‐induced cytotoxicity in drug‐resistant breast cancer cells (Fernando et al. 2023). To improve delivery and overcome pharmacokinetic barriers, nanocarrier systems, liposomal encapsulation, and polymer‐based nanoparticles are under investigation to enhance phloridzin's stability, tumor targeting, and bioavailability (Sarimahmut et al. 2022). Despite these advances, clinical translation remains constrained by a lack of human studies. Potential off‐target effects, especially those related to glucose homeostasis due to SGLT1 and SGLT2 inhibition, warrant careful evaluation. Prolonged exposure may induce hypoglycemia or gastrointestinal disturbances (Lv et al. 2024). Moreover, phloridzin's effects on immune modulation and gut microbiota are not well understood and require further study. To bridge current translational gaps, robust pharmacokinetic/pharmacodynamic (PK/PD) studies and clinical trials are needed to establish safety, optimal dosing, and efficacy in humans. Personalized approaches that consider GLUT and SGLT expression patterns across tumor types may guide patient selection and improve therapeutic outcomes. Despite extensive preclinical evidence, it is important to acknowledge that not all studies report uniformly positive outcomes for phloridzin or its derivatives. In some models, phloridzin displayed only moderate cytotoxicity or required high concentrations to achieve therapeutic effects, raising questions about its potency compared to established agents. Additionally, variability in results may stem from differences in cell line sensitivity, compound purity, and bioavailability limitations. For instance, in certain triple‐negative breast cancer lines, phloridzin showed limited efficacy unless combined with delivery systems or adjuvant drugs. These inconsistencies highlight the need for standardized protocols and further in vivo validation to clearly define the therapeutic window and optimal contexts for phloridzin application.

## 7 Conclusion

Phloridzin, a dihydrochalcone‐class natural compound derived from apples, demonstrates promising anti‐cancer properties through its anti‐oxidative, anti‐inflammatory, and metabolic regulatory effects. Its mechanisms of action involve apoptosis induction, immune system modulation, and disruption of key oncogenic signaling pathways, including the inhibition of glucose transport via GLUT1 and suppression of JAK2/STAT3 signaling, thereby impeding tumor growth and metastasis. Scientific investigations, including in vitro and in vivo studies, have consistently indicated that phloridzin can inhibit cancer cell proliferation, induce apoptosis, and interfere with glucose metabolism in various tumor models. Furthermore, its derivatives, such as fatty acid esters, exhibit enhanced bioavailability and potency, reinforcing their potential application in cancer therapeutics. The successful modification of phloridzin into more stable and efficient derivatives highlights its adaptability as a chemotherapeutic agent. Despite these advancements, phloridzin's clinical translation remains limited due to poor bioavailability, rapid metabolism, and insufficient human trials. While preclinical findings are promising, extensive pharmacokinetic and pharmacodynamic studies, as well as well‐structured clinical trials, are required to validate its therapeutic efficacy and safety profile. Future research should focus on improving its drug delivery systems, evaluating synergistic effects with existing chemotherapeutic agents, and exploring its role in personalized oncology treatments. In conclusion, phloridzin holds substantial potential as an effective and sustainable anti‐cancer agent, particularly in light of the growing global cancer burden and the demand for novel, less toxic treatment options. The continued investigation into its mechanistic pathways, bioavailability enhancement strategies, and clinical viability will be crucial in determining its place in modern oncology and its future role in integrative cancer therapy.

## Author Contributions

**Praveen Dhyani:** data curation (equal), investigation (equal), methodology (equal), project administration (equal), validation (equal), visualization (equal), writing – original draft (equal), writing – review and editing (equal). **Priyanka Sati:** data curation (equal), investigation (equal), methodology (equal), visualization (equal), writing – original draft (equal), writing – review and editing (equal). **Dharam Chand Attri:** data curation (equal), investigation (equal), methodology (equal), visualization (equal), writing – original draft (equal), writing – review and editing (equal). **Eshita Sharma:** data curation (equal), investigation (equal), methodology (equal), visualization (equal), writing – review and editing (equal). **Ruchi Soni:** data curation (equal), investigation (equal), methodology (equal), visualization (equal), writing – review and editing (equal). **Javad Sharifi‐Rad:** conceptualization (equal), data curation (equal), investigation (equal), methodology (equal), project administration (equal), supervision (equal), validation (equal), visualization (equal), writing – original draft (equal), writing – review and editing (equal). **Daniela Calina:** data curation (equal), investigation (equal), methodology (equal), project administration (equal), supervision (equal), validation (equal), visualization (equal), writing – original draft (equal), writing – review and editing (equal).

## Ethics Statement

The authors have nothing to report.

## Consent

The authors have nothing to report.

## Conflicts of Interest

The authors declare no conflicts of interest.

## Data Availability

The authors have nothing to report.
